# Supplementary figures and images for: Cellular iron governs the host response to malaria
Source: PLoS Pathog. 2023 Oct 9;19(10):e1011679. doi: 10.1371/journal.ppat.1011679 (PMC10586691; doi:10.1371/journal.ppat.1011679)

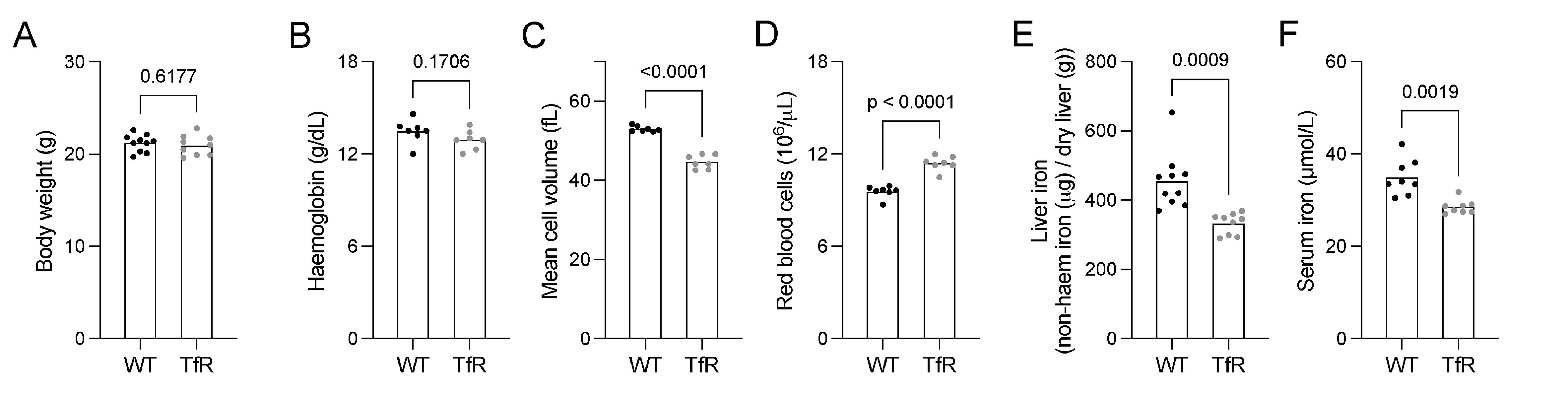

Supplement: S1 Fig — Uninfected 8–12-week-old C57BL/6 (WT) and TfrcY20H/Y20H (TfR) mice were used for characterization. A) Body weight at homeostasis. Mean, Welch’s t-test, n = 9–10. B-D) Haemoglobin (B), mean red blood cell (RBC) volume (C) and RBC count (D) at homeostasis. Mean, Welch’s t-test, n = 7. E-F) Liver iron (E) and serum iron (F) at homeostasis. Mean, Welch’s t-test, n = 8–10. (TIF) [file ppat.1011679.s001.tif]

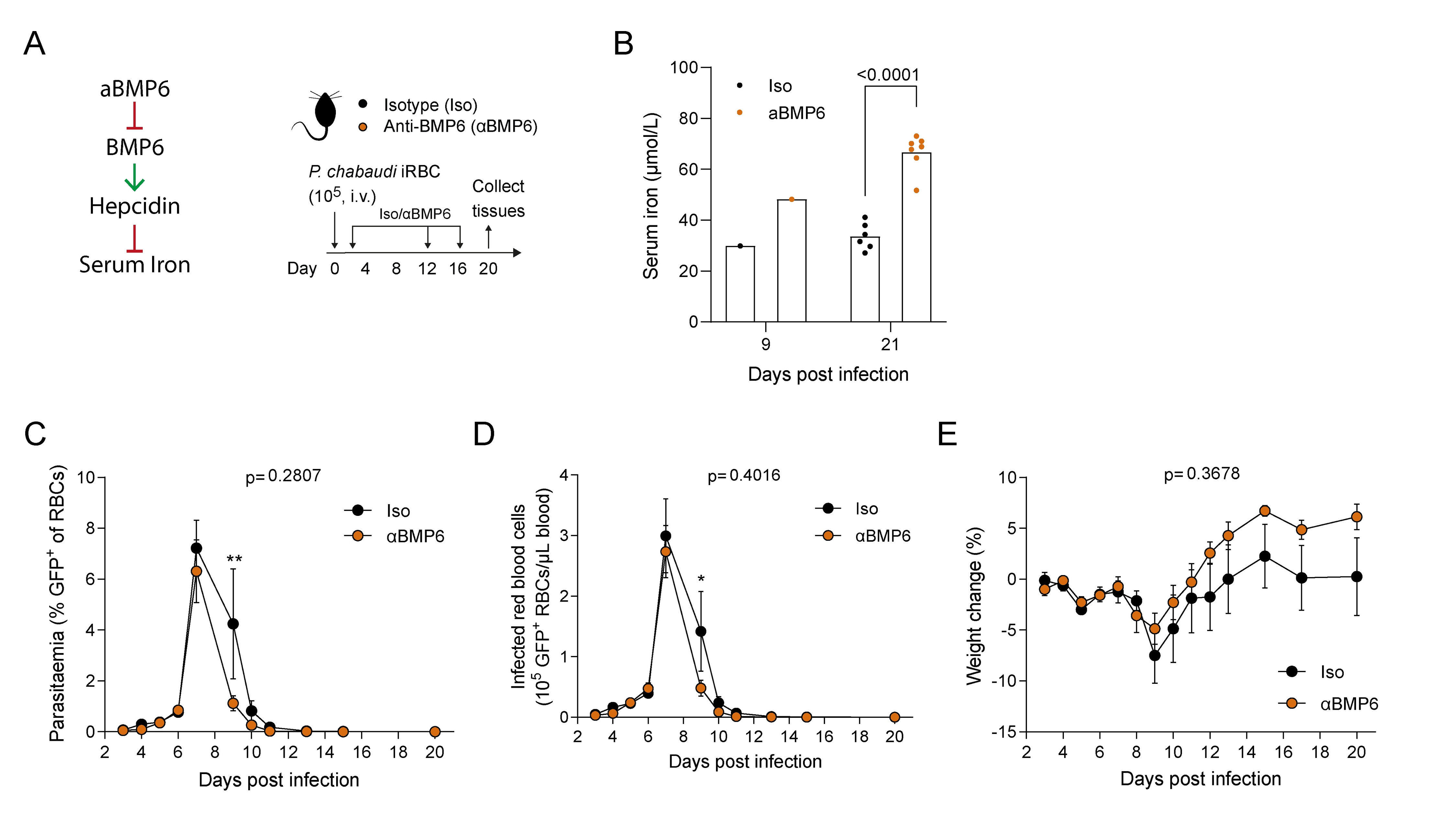

Supplement: S2 Fig — A) C57BL/6 mice were infected by intravenous (i.v.) injection of 105 P. chabaudi infected red blood cells (iRBC). A monoclonal anti-BMP-6 antibody (αBMP6) or an isotype control antibody (Iso) was administered 2, 12 and 16 days post infection (dpi). B) Serum iron measured 9 and 21 dpi in mice treated with αBMP6 or Iso. At 9 dpi, serum samples, collected through tail bleeding, were pooled for each experimental group to obtain sufficient sample for the quantification. At 21 dpi, mice were sacrificed, and serum samples collected through cardiac puncture. Mean, Welch’s t-test, n = 6–8. C-E) Parasitaemia (C), iRBC count (D) and relative change in body weight (E) were measured throughout the course of infection. Mean ± SEM, two-way ANOVA with Sidak’s multiple comparisons test, n = 6–8. (TIF) [file ppat.1011679.s002.tif]

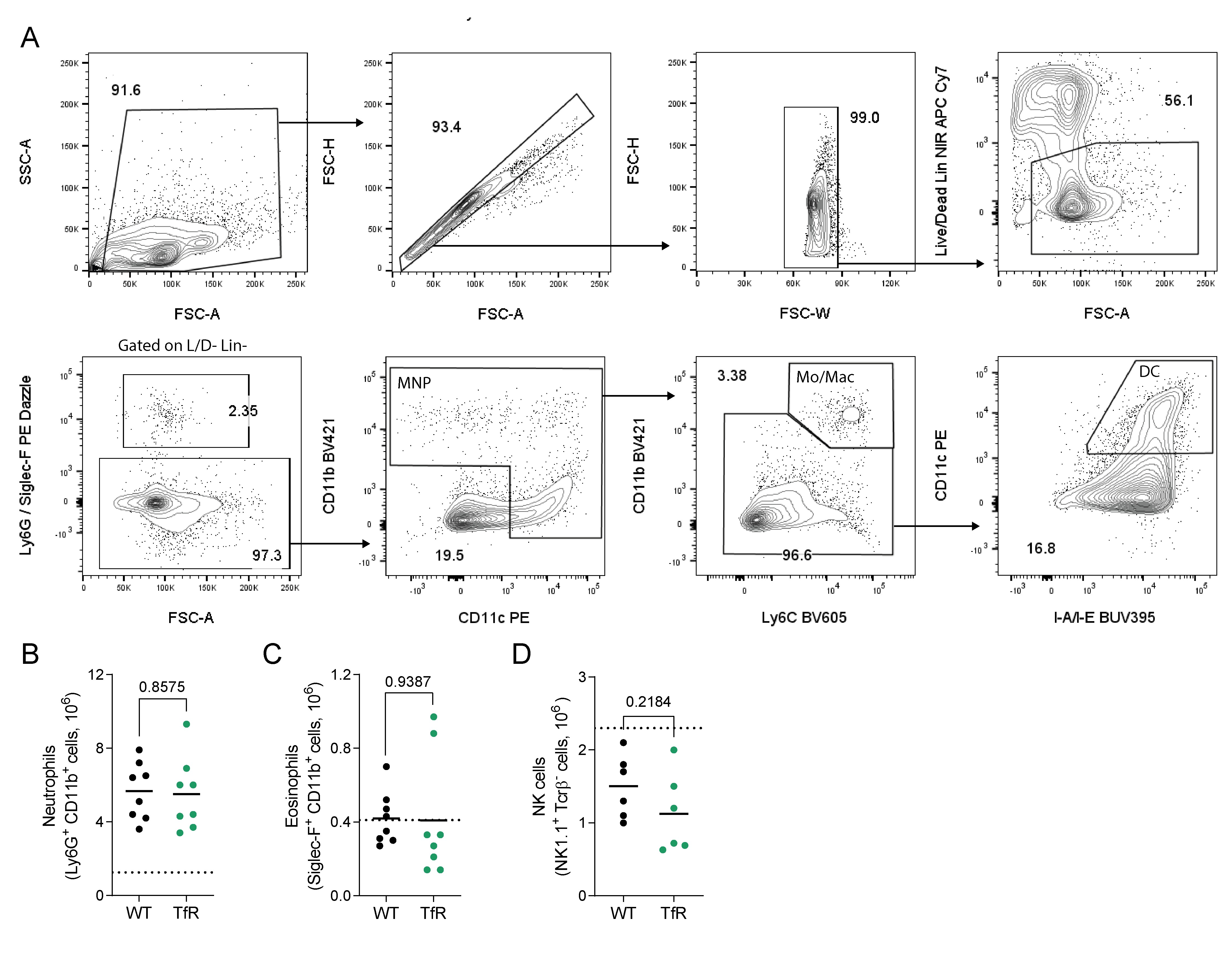

Supplement: S3 Fig — Splenic immune response of P. chabaudi infected C57BL/6 (WT) and TfrcY20H/Y20H (TfR) mice, 8 days after infection. A) Gating strategy for mononuclear phagocytes (MNP), monocytes/macrophages (Mo/Mac) and dendritic cells (DC). B-D) Absolute number of splenic neutrophils (B), eosinophils (C) and NK cells (D). Mean, Welch’s t-test, n = 6–8. (TIF) [file ppat.1011679.s003.tif]

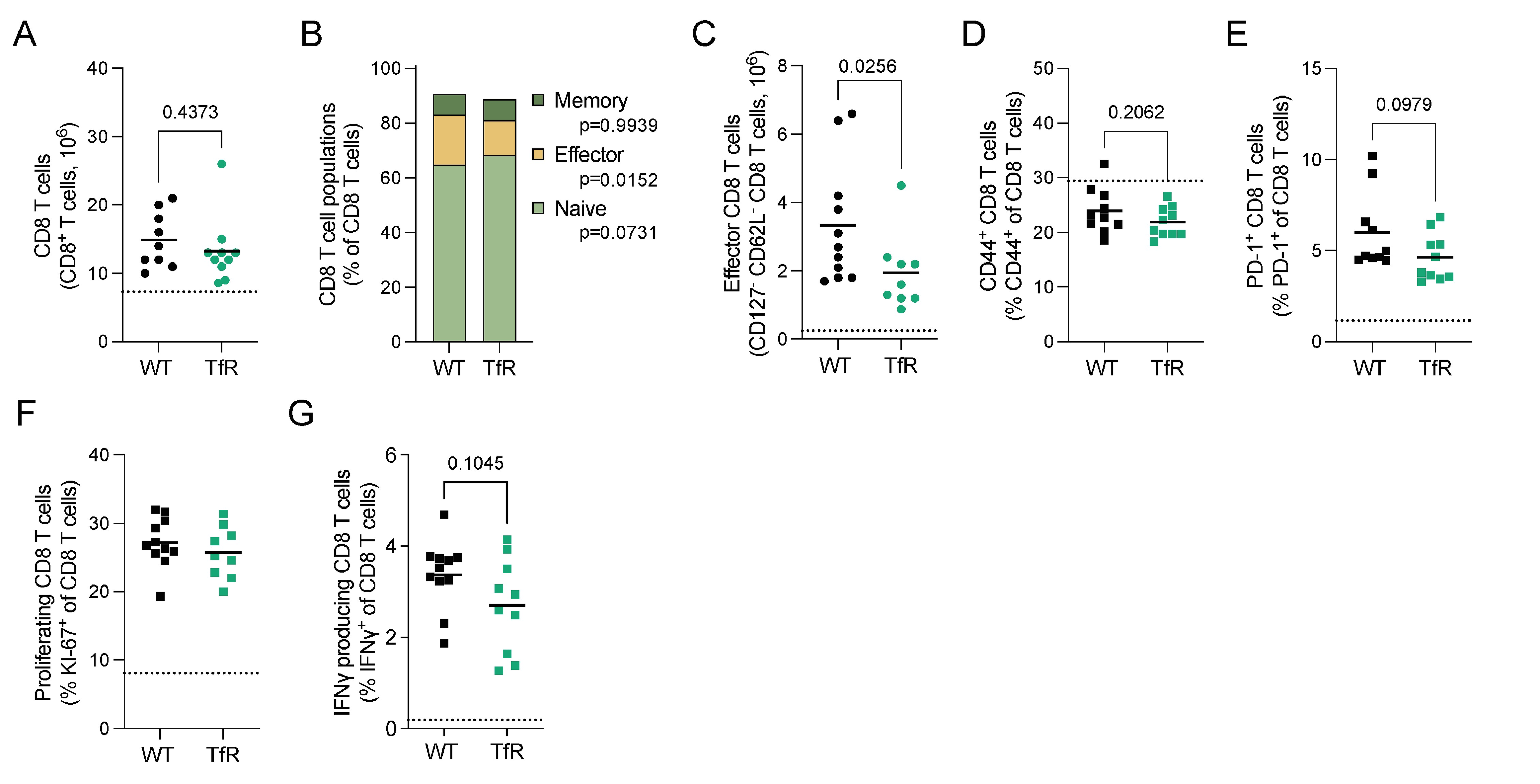

Supplement: S4 Fig — CD8+ T cells in the spleen of P. chabaudi infected C57BL/6 (WT) and TfrcY20H/Y20H (TfR) mice, 8 days after infection. A) Absolute number of CD8+ T cells. Mean, Welch’s t-test, n = 9–10. B) Proportion of naïve (CD44- CD62L+), effector (CD62L- CD127-) and memory (CD44+ CD127+) CD8+ T cells. Mean, two-way ANOVA with Sidak’s multiple comparisons test, n = 9–11. C) Absolute number of effector CD8+ T cells. Mean, Mann-Whitney test, n = 9–11. D-E) Proportion of splenic CD8+ T cells expressing markers of antigen experience CD44+ (D) and PD-1+ (E). Mean, Welch’s t-test n = 10. F) Proportion of proliferating (KI-67+) CD8+ T cells. Mean, Welch’s t-test n = 9–11. G) Proportion of IFNγ producing CD8+ T cells, detected by intracellular cytokine staining. Mean, Welch’s t-test n = 10–11. Dotted line represents uninfected mice. (TIF) [file ppat.1011679.s004.tif]

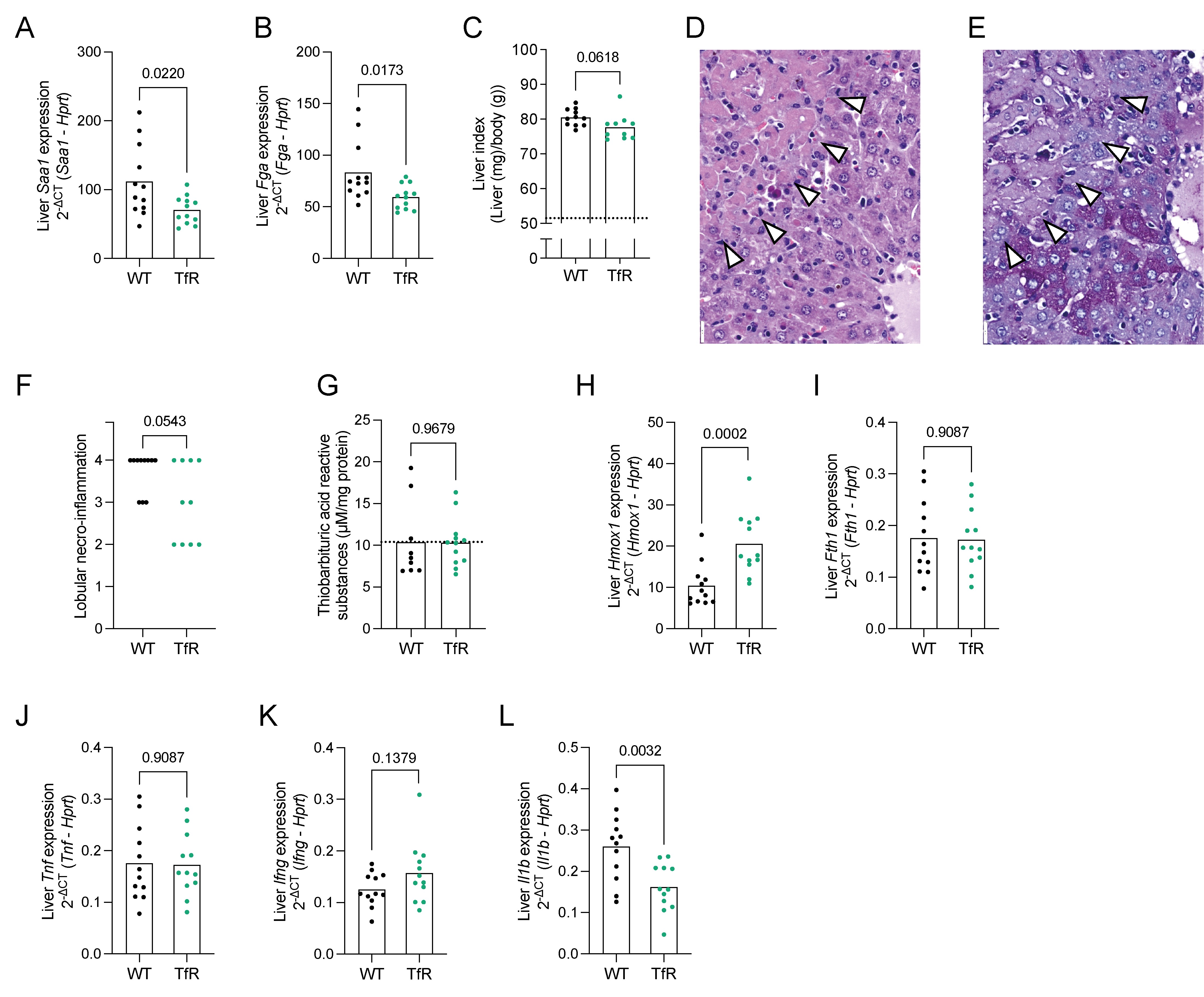

Supplement: S5 Fig — Hepatic response of P. chabaudi infected C57BL/6 (WT) and TfrcY20H/Y20H (TfR) mice, 8 days after infection. A-B) Liver gene expression of Saa1 (A) and Fga (B). Mean, Welch’s t-test, n = 12. C) Liver index. Mean, Welch’s t-test, n = 10–11. D-E) Higher magnification depiction of H&E (D) and PAS (E) stained liver sections from a representative P. chabaudi infected WT mouse. The arrowheads indicate areas of confluent necrosis, featuring lobular disarray, lympho-histiocytic inflammation, acidophil body formation, and glycogen depletion. Original magnification 200X, scale bar 20 μm. F) Blinded scoring of lobular necro-inflammatory activity. Mann-Whitney test, n = 10–11. G) Hepatic malondialdehyde (MDA), quantified as an indirect measurement or ROS, using a thiobarbituric acid reactive substances assay. Mean, Welch’s t-test, n = 10–12. H-L) Liver gene expression of Hmox1 (H), Fth1 (I), Tnf (J), Ifng (K) and Il1b (L). Mean, Welch’s t-test on untransformed (H, I, J & L) or log transformed data (K), n = 12. (TIF) [file ppat.1011679.s005.tif]
