# Supplementary material for: Cellular iron governs the host response to malaria
Source: PLoS Pathog. 2023 Oct 9;19(10):e1011679. doi: 10.1371/journal.ppat.1011679 (PMC10586691; doi:10.1371/journal.ppat.1011679)
Supplement: Attachment — Submitted filename: 134043_1_rebuttal_2720146_rx02kc.pdf [file ppat.1011679.s006.pdf]

Full Revision

Review COMMONS

Manuscript number: RC-2023-01870

Corresponding author(s): Hal Drakesmith; Sarah Wideman

1. General Statements [optional]

This section is optional. Insert here any general statements you wish to make about the goal of the study or about the reviews.

2. Point-by-point description of the revisions

This section is mandatory. Please insert a point-by-point reply describing the revisions that were already carried out and included in the transferred manuscript.

Reviewer #1

Evidence, reproducibility and clarity (Required):

In this paper by Wideman et al, the authors seek to determine the role of cellular iron homeostasis in the pathogenesis of murine malaria.

The authors to attempt to disentangle the effects of anemia from that of cellular iron deficiency. The authors elegantly make use of a murine model of a rare human mutation in the transferrin receptor. This mutation leads to decreased receptor internalization and decreased cellular iron, but otherwise healthy mice. Using this model, the authors use a *P. chabaudi* infection model and show an increase in pathogen burden and a decrease in pathology. They show in some detail that the immune response to *P. chabaudi* infection is blunted, both T and B-cell responses are attenuated in the $TfR^{Y20H/Y20H}$ model, and the block in proliferation can be rescued by exogenous iron supplementation. They also show that decreased cellular iron attenuates liver pathology through potentially multiple mechanisms.

Minor comments:

- The peak of parasitemia is relatively low (approx..3%) compared to other published studies (e.g. PMID: 22100995, 16714546, 31110285) where the peak in C57BL/6 mice reached 25 - 40%. Can the authors account for this low parasitemia?

  Response: We thank the reviewer for their constructive comments and appreciate that they are highlighting this important point. It has previously been shown (PMID: 23217144, 23719378) that mosquito-transmission of *P. chabaudi* leads to significantly lower parasitaemia (<10%), compared to infection with serially blood-passaged parasites. This lower parasitaemia is more in line with parasitaemias observed in human infection. The studies referred to by Reviewer #1 were performed with parasites that had been serially

blood-passaged (PMID: 22100995, 16714546, 31110285), which makes them more virulent. Hence, the parasitaemias observed in our study are in line with what is expected for recently mosquito-transmitted parasites. This point has been further clarified in the manuscript (line **104-108**):

"Recently mosquito-transmitted parasites were used to mimic a natural infection more closely, as vector transmission is known to regulate Plasmodium virulence and alter the host's immune response (48-50). Consequently, parasitaemia is expected to be significantly lower upon infection with recently mosquito-transmitted parasites, compared to infection with serially blood-passaged parasites that are more virulent (48,49)."

- Figure 1K - At homeostasis, serum iron is low in TfR mice however increases to significantly higher than the WT mice at 8 days post infection. Do the authors have an explanation on why these dramatic changes in serum iron are seen?

  Response: During malaria infection, RBC lysis releases haem and iron into circulation, which leads to an increase in serum iron levels. This effect is observed in both wild-type and *Tfrc*$^{Y20H/Y20H}$ mice infected with *P. chabaudi* (Supplementary Figure 1F & Figure 1K). However, the significantly higher serum iron levels observed in infected *Tfrc*$^{Y20H/Y20H}$ mice can likely be explained by their decreased capacity for transferrin receptor-1 mediated iron uptake, leading to relatively slower uptake and storage of circulating transferrin-bound iron into tissues. This has been clarified in the manuscript (line **142-143**):

  "The elevated serum iron observed in infected Tfrc$^{Y20H/Y20H}$ *mice was consistent with their restricted capacity to take up transferrin-bound circulating iron into tissues."*

- Figure S3 - Is it surprising that no effects on splenic neutrophils are seen? Were neutrophils quantified at any other point? These would also be expected to have a role in both the control of malaria infection and on any pathology.

  Response: We thank the reviewer for raising this interesting question. It is known that neutrophils can be sensitive to cellular iron deficiency (PMID: 36197985) and that neutrophils can play an important part in malaria infection (PMID: 31628160). However, the magnitude and significance of the neutrophil response to recently mosquito-transmitted *P. chabaudi* parasites has not been thoroughly investigated. A recent study demonstrated that monocytes and macrophages may be more important than granulocytes in the early response to recently mosquito-transmitted *P. chabaudi* infection (PMID: 34532703).

  Moreover, we performed neutrophil quantifications in our initial experiments and found that the splenic neutrophil response was not altered in *Tfrc*$^{Y20H/Y20H}$ mice eight days after infection. Additionally, no neutrophil infiltration was observed in the liver of either genotype upon *P. chabaudi* infection. In light of these findings, we did not characterise the neutrophil response further, as it appeared unlikely that neutrophils were the principal causal agent of either the altered immunity or pathology, in this context. However, we agree with the reviewer that larger

question of whether neutrophil iron plays a role in the pathology of malaria is an interesting open question which we hope future studies can elucidate.

A section was added to the discussion to address the role of innate immune cells in our model (line **354-363**):

"The inhibited innate immune response to P. chabaudi in Tfrc$^{Y20H/Y20H}$ mice likely contributed to both the increased pathogen burden and the decreased liver pathology. Splenic MNPs are important for controlling parasitaemia (34,35,72), but MNPs are also vital for maintaining tissue homeostasis and preventing tissue damage in malaria (43,73). Although other innate cells, such as neutrophils, NK cells and γδT cells are an important part of the immune response to malaria, only the MNP response was distinctly impaired in Tfrc$^{Y20H/Y20H}$ mice. Notably, neutrophils are known to be sensitive to iron deficiency (16,74) and to affect both immunity and pathology in malaria (75,76). However, in the context of recently mosquito-transmitted P. chabaudi it appears that monocytes and macrophages, rather than granulocytes, may be particularly important for parasite control and tissue homeostasis (43,72)."

<u>Changes to the text:</u>

- Fig S1EandF - Please add to the figure legend that these were measured at homeostasis.

  Response: This clarification has been added to the legend of Supplementary Figure 1 (line **954-957**).

- Figure 3 - In the legend, H and I are the wrong way around.

  Response: The legend of Figure 3 has been corrected accordingly (line **888-890**).

- Figure 4 - please add the units of concentration of FeSO4 to all panels

  Response: The units of concentration for $FeSO_4$ and AFeC have been added to all panels of Figure 4 and 6, respectively.

- Line 246 - The authors state: "there was some evidence of decreased malaria-induced hepatomegaly" however there is no significant difference between WT and TfR mice and both show significant hepatomegaly. I feel that this line should be reworded.

  Response: The sentence (line **252-254)** has been reworded as follows:

  "*Furthermore, while both genotypes developed malaria-induced hepatomegaly, there was a trend toward less severe hepatomegaly in Tfrc$^{Y20H/Y20H}$ mice (Figure S5C)."*

<u>Significance (Required):</u>

This work is one of the first to attempt to define the requirements for cellular iron in malaria infection. This is a difficult topic, as infection and associated inflammation and the red blood cell

destruction caused by malaria all have complex effects on iron within the body. This study fits well with previous observations showing that anemia can be protective as it both prevents parasite growth and limit immunopathology. This work advances the field by demonstrating a cell intrinsic role for iron in malaria infection. There is a broad possible audience for this work, including malaria researchers, immunologists and people interested in the role or iron, both at a cellular level and systemically.

Reviewer #2

Evidence, reproducibility and clarity (Required):

In this manuscript, the authors have studied the role of iron deficiency in the host response to *Plasmodium* infection using a transgenic mouse model that carries a mutation in the transferrin receptor. They show that restricted cellular iron acquisition attenuated *P. chabaudi* infection-induced splenic and hepatic immune responses which in turn mitigated the immunopathology, even though the peak parasitemia was significantly high in the mutant mice. Interestingly, the course of parasite infection doesn't seem to be affected in the mutant mice compared to the wildtype mice despite the induction of poor immune responses. The authors show that the decreased cellular iron uptake broadly impact both innate and adaptive components of the immune system. Conversely, free iron supplementation restored the immune cell functions.

- The study is well performed, and the manuscript is well written. However, the authors should show how conserved the role of cellular iron is across other rodent malaria parasite species at least with *P. yoelii* or *P. berghei* blood stage infection models. This question becomes critical to address in order to understand broad relevance to human malaria infections where both the host and parasites are genetically diverse.

  Response: We thank the reviewer for appreciating our study and for the thoughtful comments. We agree with the reviewer that the diverse genetic background of both parasites and hosts makes it difficult to draw broad conclusions about human malaria infection from animal studies performed in a laboratory setting. The recently mosquito-transmitted *P. chabaudi chabaudi* AS blood-stage infection model replicates many key features of mild to moderate malaria infection in humans, such as low parasitaemia, anaemia, cyto-adhesive sequestration in microvasculature, and self-resolving immunopathology. Importantly, the immune response elicited by recently mosquito-transmitted parasites also more closely mimics the immune response to a natural infection (PMID: 23719378). Therefore, we consider the recently mosquito-transmitted *P. chabaudi chabaudi* AS model as the most relevant to answer our particular research questions.

  Furthermore, specific pathogen-free parasitised erythrocyte stabilates made from recently mosquito-transmitted *P. berghei* or *P. yoelii* parasites are unfortunately not readily accessible (e.g. through the European Malaria Reagent Repository), in contrast to *P. chabaudi*.

Consequently, preparing and characterising recently mosquito-transmitted strains to perform the experiments suggested by the reviewer would require a substantial amount of additional time and labour, which we deem out of scope for this study.

In the design of our model we have also taken care to minimise the effects of anaemia, something which would be difficult or impossible to achieve using serially blood passaged *P. yollii* or *P. berghei* parasites. Both *P. yoelii* and *P. berghei* merozoites preferentially invade immature RBCs (PMID: 34322397) making readouts such as parasitaemia far more sensitive to small variations in erythropoietic output. In addition, the extensive RBC destruction caused by most serially blood-passaged murine *Plasmodium* strains would likely exaggerate any erythropoietic impairment caused by the *Tfrc*$^{Y20H/Y20H}$ mutation.

Although we strongly believe that the chosen mouse model of malaria is the most appropriate for our study, ultimately, no mouse model can replicate all features of human malaria infection. Inevitably, the direct relevance of animal studies for human infection will always be somewhat opaque. Hence, we respectfully disagree with the reviewer that repeating the experiments with additional murine malaria parasite species would allow us to extrapolate conclusions about human malaria infection. Such experiments would also conflict with the 3Rs principles that govern work with animals in the UK (https://nc3rs.org.uk/). Especially, because most strains of *P. yoelii* and *P. berghei* cause severe or non-resolving infections and have a significant negative impact on animal welfare.

In our opinion, the logical continuation of this study must be to utilise the insights from our research to inform future human studies on the relationships between iron deficiency and malaria-related immunopathology. However, we agree that this is an important topic and have added a section addressing the broad relevance of our findings to the discussion (line **393-396**):

"It remains to be seen what the broader importance of cellular iron is in human malaria infection, in particular within the diverse genetic context of both humans and parasites found in malaria endemic regions. Murine models of malaria are useful in providing hypothesis-generating results, but such findings ultimately ought to be confirmed and developed further through studies in human populations."

- Since, restricted cellular iron uptake mitigates the immunopathology, the authors should explore whether this could also relieve the cerebral malaria condition that is caused by the hyper inflammation in the brain. They should use the *P. berghei* ANKA parasite strain which causes t cerebral malaria in mice. I think would increase impact of the paper.

Response: Although we agree that this would be an interesting line of inquiry, we think that it is outside of the scope of this study, which predominantly aims to characterise and study the effects of cellular iron deficiency in host cells, particularly immune cells, during mild to moderate malaria infection. The severe pathology underlying cerebral malaria differs greatly

from that of a self-resolving blood-stage infection. Furthermore, the relevance to human cerebral malaria of the *P. berghei* ANKA model is controversial within the field (PMID: 21288352) and as a severe infection its use would again conflict with the 3Rs principles.

Minor comments:

- Line 222: repeating word, "iron iron-supplemented...."

  Response: The sentence has been corrected (line **228**).

- Figure 3C, S4C & S5F: Why Mann-Whitney test is performed in these particular graphs, whereas rest of the two groups comparison were done using Welch's test? The authors should clearly mention this in the methods section.

  Response: We apologise if this was unclear in the manuscript. We routinely tested all our datasets for normality to identify the appropriate tests for each dataset. In case of the graphs shown in figure 3C, S4C and S5F, the dataset did not pass the D'Agostino-Pearson normality test and we therefore applied a non-parametric test (i.e. Mann-Whitney), in contrast to the other datasets that passed the test for normal or lognormal distribution. This has been further clarified in the method section (line **581-586**):

  "*The D'Agostino-Pearson omnibus normality test was used to determine normality/lognormality. Parametric statistical tests (e.g. Welch's t-test) were used for normally distributed data. For lognormal distributions, the data was log-transformed prior to statistical analysis. Where data did not have a normal or lognormal distribution, or too few data points were available for normality testing, a nonparametric test (e.g. Mann-Whitney test) was applied*."

- Have authors explored whether gamma-delta T cell responses are affected in the mutant mouse strain compared to wildtype mice as they are one of the early responders and the key cytokine producing cells against the *Plasmodium* blood stage infection.

  Response: We thank the reviewer for this valuable comment. We briefly explored the role of γδT cells, but did not observe a significant difference in splenic γδT cell numbers between wild-type and $Tfrc^{Y20H/Y20H}$ mice, eight days post-infection (**Reviewer Figure 1**). It is of course possible that γδT cell numbers were affected at an earlier stage, or that γδT cell function (e.g. cytokine production) was affected by cellular iron deficiency during *P. chabaudi* infection. However, γδT cells may also be less sensitive to cellular iron deficiency than conventional T cells, as has been previously demonstrated for developing T cells (PMID: 7957580).

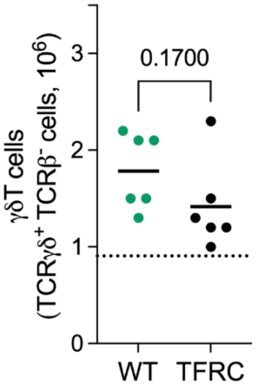

Reviewer figure 1. Decreased cellular iron uptake did not alter the splenic γδT cell response to *P. chabaudi,* eight days after infection. Mean, Welch's t-test, n=6.

A section was added to the discussion to address the role of innate immune cells in our model (line **354-363**):

"The inhibited innate immune response to P. chabaudi in Tfrc$^{Y20H/Y20H}$ mice likely contributed to both the increased pathogen burden and the decreased liver pathology. Splenic MNPs are important for controlling parasitaemia (34,35,72), but MNPs are also vital for maintaining tissue homeostasis and preventing tissue damage in malaria (43,73). Although other innate cells, such as neutrophils, NK cells and γδT cells are an important part of the immune response to malaria, only the MNP response was distinctly impaired in Tfrc$^{Y20H/Y20H}$ mice. Notably, neutrophils are known to be sensitive to iron deficiency (16,74) and to affect both immunity and pathology in malaria (75,76). However, in the context of recently mosquito-transmitted P. chabaudi it appears that monocytes and macrophages, rather than granulocytes, may be particularly important for parasite control and tissue homeostasis (43,72)."

Significance (Required):

Overall, the study provides novel insights into the role of iron in the immune response to Plasmodium blood stage infection using a rodent malaria model and the interplay of infection, immunity and the development of pathology. As such it is an important study.

Reviewer #3

Evidence, reproducibility and clarity (Required):

Herein Wideman provide novel and important evidence on the role of iron availability for mounting an efficient immune response in a malaria infection model. They employed TfRC Y201H/Y201H mice which develop iron deficiency due to impaired cellular ingestion of transferrin bound iron. They found that those mice develop higher peak parasitemia after vector borne exposure to Pl.

chabaudi chabaudi which was paralleled by an impaired immune response as reflected by altered CD4 cell activation, reduced IFN-g formation or reduced B-cell responsiveness. Those deficiencies could be re-covered upon ex vivo iron supplementation pointing to the importance of iron availability for mounting-CD4+ and B-cell specific anti-plasmodial immune responses at the initial phase of infection. However, TFRC mutated mice were able to clear infection over time in a comparable fashion to wt mice.
This excellent study is important in convincingly showing (by employing high quality immunological analyses) the importance of cellular iron deficiency on immune responses in an infection model of general interest. It also indicates that overwhelming immune response as seen in wt mice is associated with organ damage over time.

Minor comments:

- The authors should discuss why and how TFRC mutated mice were able to control infection over time in a comparable fashion as wt mice although peak parasitemia was significantly higher?

  Response: We thank the reviewer for the helpful feedback on our study and for posing this interesting question. It does indeed appear as if the immune response, while significantly inhibited in the *Tfrc*$^{Y20H/Y20H}$ mice, is still sufficient to clear the infection. It is plausible that the early cell-mediated immune response is inhibited to the degree that parasite control is impaired, resulting in higher peak parasitaemia in *Tfrc*$^{Y20H/Y20H}$ mice. In contrast, parasite clearance is comparable and contemporary in both genotypes. Based on the fact that parasite clearance occurs at a time when a substantial adaptive immune response is expected to emerge, we hypothesize that this significantly contributes to pathogen clearance. Thus, it seems likely that the humoral response in *Tfrc*$^{Y20H/Y20H}$ mice, even if inhibited, may still be effective enough to clear the parasites and prevent recrudescence.

  As malaria infection progresses, RBC loss and increasing anaemia also contributes to limiting exponential parasite growth. This occurs more or less equally in both genotypes, but it could be particularly important for parasite control in the *Tfrc*$^{Y20H/Y20H}$ mice that have an inhibited immune response.

  We have added a section to the discussion to address this (line **380-386**):

  "Despite the higher peak parasitaemia in Tfrc$^{Y20H/Y20H}$ *mice, both genotypes were able to clear P. chabaudi parasites at a comparable rate and prevent recrudescence. It follows that even a weakened humoral immune response appears to be sufficient to control P. chabaudi infection. However, our study did not investigate the effects of immune cell iron deficiency on the formation of long-term immunity, which may have been more severely affected. The impaired GC response, in particular, suggests that iron deficiency could counteract the formation of efficient immune memory to subsequent malaria infections."*

- The authors and others have previously shown (Frost J et al. Sci Adv 2022, Hoffmann et al. EBioMedicine 2021) that iron deficiency results in reduced neutrophil numbers in different infection models. This could also have contributed to the observed effect in initial infection control but may have also been linked altered histopathology seen in Figure 7. However, no mention of neutrophil numbers in this model is made. It would be important if the authors could provide information on neutrophil numbers (only if this analysis has been already performed) and discuss this issue in association with their observation.

  Response: We appreciate that the reviewer has brought attention to this important topic. As they mention, iron deficiency can have a negative impact on the neutrophil response (PMID: 36197985, 34488018) but it can also cause a maladaptive excessive neutrophil response due to failed adaptive immunity (PMID: 33665641). In this study, we show that there is no difference in splenic neutrophil numbers between wild-type and *Tfrc*$^{Y20H/Y20H}$ mice, eight days after *P. chabaudi* infection (Figure S3B). Moreover, the histopathologists detected no liver neutrophil infiltration in either genotype, but rather observed infiltration of mononuclear leukocytes upon *P. chabaudi* infection. Hence, it appears unlikely that neutrophils were a major contributor to differences in either immunity or pathology in this specific context. However, we cannot definitively rule out that neutrophil numbers were affected earlier in the infection or that neutrophil function was impaired due to cellular iron deficiency.

  A section was added to the discussion to address the role of innate immune cells in our model (line **354-363**):

  "The inhibited innate immune response to P. chabaudi in Tfrc$^{Y20H/Y20H}$ *mice likely contributed to both the increased pathogen burden and the decreased liver pathology. Splenic MNPs are important for controlling parasitaemia (34,35,72), but MNPs are also vital for maintaining tissue homeostasis and preventing tissue damage in malaria (43,73). Although other innate cells, such as neutrophils, NK cells and γδT cells are an important part of the immune response to malaria, only the MNP response was distinctly impaired in Tfrc*$^{Y20H/Y20H}$ *mice. Notably, neutrophils are known to be sensitive to iron deficiency (16,74) and to affect both immunity and pathology in malaria (75,76). However, in the context of recently mosquito-transmitted P. chabaudi it appears that monocytes and macrophages, rather than granulocytes, may be particularly important for parasite control and tissue homeostasis (43,72)."*

- In addition, alternative mechanism leading to immune tolerance and reduced tissue damage such as induction of heme oxygenase-1, which is also affected by systemic iron availability, should be discussed.

  Response: An addition was made to the results section and to Figure S5 to address this reviewer comment (line **269-274**):

"In addition, we measured the expression of two genes that are known to have a hepatoprotective effect in the context of iron loading in malaria: Hmox1 (encodes haemoxygenase-1) and Fth1 (encodes ferritin heavy chain). Liver gene expression of Hmox1 was higher in Tfrc$^{Y20H/Y20H}$ mice, while the expression of Fth1 did not differ between genotypes, eight days after infection (Figure S5H-I). Thus, the higher expression of Hmox1 may have contributed to the hepatoprotective effect in Tfrc$^{Y20H/Y20H}$ mice."

A relevant sentence was also added to the discussion (line **313-318**):

"For example, HO-1 plays an important role in detoxifying free haem that occurs as a result of haemolysis during malaria infection, thus preventing liver damage due to tissue iron overload, ROS and inflammation (62). Interestingly, infected Tfrc$^{Y20H/Y20H}$ mice had higher expression of Hmox1, but levels of liver iron and ROS comparable to that of wild-type mice. Consequently, this may be indicative of increased haem processing that could have a tissue protective effect"

Significance (Required):

Important and intersting study highlighting the central role of iron homeostasis for immune repsonse to infection. General interest because iron deficiency has high prevalence in areas with high enedemic burden of infection

Reviewer's expertise: infectious disease, immunity, iron homeostasis-- both basic science and clincal expertise (more than 300 peer reviewed publications on these topcis)
